# Biomarkers for febrile urinary tract infection in children

**DOI:** 10.3389/fped.2023.1163546

**Published:** 2023-05-09

**Authors:** Nader Shaikh, Marcia Kurs-Lasky, Hui Liu, Vinod Rajakumar, Heba Qureini, Isabella O. Conway, Matthew C. Lee, Sojin Lee

**Affiliations:** Division of General Academic Pediatrics, Children’s Hospital of Pittsburgh of UPMC, University of Pittsburgh School of Medicine, Pittsburgh, PA, United States

**Keywords:** urinary tract infection (UTI), diagnostic accuracy, biomarker, infectious disease, microbiome

## Abstract

**Background:**

The current reference standard for pediatric urinary tract infection (UTI) screening, the leukocyte esterase (LE) dipstick test, has suboptimal accuracy. The objective of this study was to compare the accuracy of novel urinary biomarkers to that of the LE test.

**Methods:**

We prospectively enrolled febrile children who were evaluated for UTI based on their presenting symptoms. We compared the accuracy of urinary biomarkers to that of the test.

**Results:**

We included 374 children (50 with UTI, 324 without UTI, ages 1–35 months) and examined 35 urinary biomarkers. The urinary biomarkers that best discriminated between febrile children with and without UTI were urinary neutrophil gelatinase–associated lipocalin (NGAL), IL-1β, CXCL1, and IL-8. Of all examined urinary biomarkers, the urinary NGAL had the highest accuracy with a sensitivity of 90% (CI: 82–98) and a specificity of 96% (CI: 93–98).

**Conclusion:**

Because the sensitivity of the urinary NGAL test is slightly higher than that of the LE test, it can potentially reduce missed UTI cases. Limitations of using urinary NGAL over LE include increased cost and complexity. Further investigation is warranted to determine the cost-effectiveness of urinary NGAL as a screening test for UTI.

## Introduction

Diagnosis of urinary tract infection (UTI) relies on the urine culture, which typically requires 48–72 h. This forces clinicians to rely on bedside screening tests to determine which children need treatment with antimicrobials. However, neither the leukocyte esterase (LE) test nor the leukocyte count (WBC) obtained using conventional urine microscopy is sufficiently specific to serve as a screening test. LE and WBC were found to have a sensitivity and specificity of 79% and 87% and 74% and 86%, respectively (1). The low specificity of these tests results in the inappropriate use of antimicrobials (2); approximately 50% of children prescribed antimicrobials for UTI were eventually proven not to have a UTI (3). More specific markers for UTI would reduce unnecessary antimicrobial use. In addition, the low sensitivity of the available screening tests leads to missed UTIs, particularly UTIs caused by organisms other than *Escherichia coli*, where the sensitivity of the current screening tests approaches 50% (4).

Compared to urinary leukocytes, which can be elevated in various conditions, urinary proteins intricated in the host's response to a pathogen might be better suited to serve as screening tests for UTI. In a previous case–control study, urinary neutrophil gelatinase–associated lipocalin (NGAL) was more accurate than the available point-of-care tests currently used to screen children for UTI. However, because accuracy estimates are often inflated in case–control studies (5), we revisited the accuracy of urinary NGAL in our ongoing prospective study partly aimed at identifying better markers to screen for UTI in young febrile children.

## Materials and methods

Between June 2019 and May 2020, we enrolled consecutive children in a prospective study at the Children's Hospital of Pittsburgh Emergency Department. Children were included if they were between 1 month and 3 years of age, had a fever (temperature of ≥38°C) within 24 h of presentation, and had a urine sample collected via a catheter. We excluded children who had received systemic antimicrobials or corticosteroids within 72 h of enrolment, had other concurrent systemic bacterial infections, were immunodeficient, had a neurogenic bladder, or had major genitourinary abnormalities. Of note, routine practice at our institution is to perform bladder catheterization on all non–toilet-trained children with suspected UTI. The study was approved by the University of Pittsburgh Institutional Review Board, and no consent was required from the parents of participating children. We stored 0.2 ml aliquot of urine in a cryovial without preservatives at −80°C within 1 h of sample collection. However, if a delay was anticipated, samples were stored in a specimen refrigerator until processing.

### Protein measurement

We assessed 35 candidate markers. These markers were measured in urine using a commercially available 34-plex plate (ProcartaPlex, EPX340-12167-901, Thermo Fisher Scientific; see Table 2 for a listing of these markers) and Bioporto NGAL Human ELISA kit (KIT036RUO; Bioporto). On each plate, we included duplicates and control samples.

We first ran the 34-plex plate by following the manufacturer’s recommendations. We used 50 μl of the sample and processed samples in five batches. Plates were read on Bio-Plex MAGPIX instrument (Bio-Rad) after performing the recommended calibration and verification steps using MAGPIX Performance Verification Kit (Bio-Rad, MPX-PVER-K25) and MAGPIX Calibration Kit (Bio-Rad, MPX-CAL-K25). Data were processed using the Bio-Plex Manager Software (version 6.1, Bio-Rad). NGAL ELISA was performed per the manufacturer's instructions and using a 500-fold dilution; plates were read using BenchmarkPlus (Bio-Rad). We ran the NGAL ELISA's after the 34-plex plate because freeze-thaw cycles have little effect on NGAL values (6). We did not normalize biomarker values to urine creatinine because we previously found that this decreased accuracy (7–10). The laboratory technician was blinded to the target condition.

### Target condition and reference standards

Urinary tract infection was the target condition examined. The reference standard for diagnosis of UTI was the urine culture. We defined UTI as the growth of at least one organism at ≥10,000 colony-forming units/milliliter (CFU/ml) from a catheterized specimen.

### Statistical methods

We used the *t*-test to compare levels of the respective urinary markers in those with and those without UTI. To account for multiple comparisons, we converted *p*-values to *q*-values using the Benjamini–Hochberg correction (11). For each marker, we calculated the area under the receiver operating characteristic (ROC) curve and the sensitivity and specificity values that would maximize the Youden index (*J*), where *J* is the sensitivity plus the specificity minus 1. Also, for each marker, we calculated the sensitivity and specificity values that would minimize the distance (D) from the ROC curve to the point (0,1), i.e., where 1 minus specificity equals 0 and sensitivity equals 1. We used linear regression to explore the effect of covariates (age, gender, race, and isolated organisms) on marker accuracy. We evaluated the accuracy of combinations of biomarkers in predicting UTI using a procedure that creates a decision tree model (SAS PROC HPSPLIT). Entropy criterion was specified for the GROW statement and cost complexity for the PRUNE statement.

## Results

**Figure 1:**
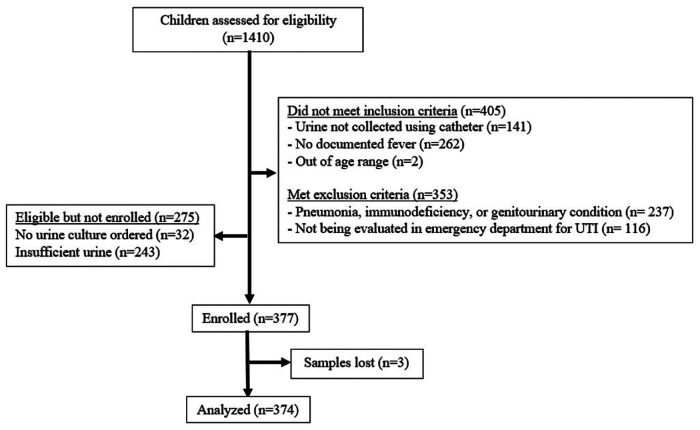
Flow of patients in the study.

Figure 1 shows the flow of patients in the study, 652 of whom were eligible for enrolment. Table 1 describes the demographic characteristics of the 374 children included in the analysis. The prevalence of UTI was 13.4% (50/374). As expected, children with UTI were mostly female (88.0%).

**Table 1:** Demographic and clinical characteristics of the children included.

Characteristic	No UTI^a^ (*N* = 324)	UTI^a^ (*N* = 50)
	Number (%)	Number (%)
Age (months)		
1–11	156 (48.1)	22 (44.0)
12–23	124 (38.3)	21 (42.0)
24–35	44 (13.6)	7 (14.0)
Sex/circumcision		
Female	234 (72.2)	44 (88.0)
Uncircumcised male	16 (4.9)	4 (8.0)
Circumcised male	54 (16.7)	2 (4.0)
Unknown if circumcised	20 (6.2)	0 (0)
Race		
White	213 (65.7)	41 (82.0)
Black	75 (23.1)	4 (8.0)
Asian	11 (3.4)	3 (6.0)
Multiracial	2 (0.6)	0 (0)
Other	3 (0.9)	0 (0)
Not reported	20 (6.2)	2 (4.0)
Maximum reported temperature (°C)		
≥39	206 (63.6)	36 (72.0)
38–39	118 (36.4)	14 (28.0)
Leukocyte esterase		
Negative	312 (96.3)	6 (12.0)
Trace	2 (0.6)	1 (2.0)
1+	7 (2.2)	11 (22.0)
2+	1 (0.3)	9 (18.0)
3+	2 (0.6)	23 (46.0)
WBC		
<5 hpf	49 (15.1)	0 (0)
≥5 hpf	5 (1.5)	6 (12.0)
<10 mm^3^	259 (80.0)	10 (20.0)
≥10 mm^3^	11 (3.4)	34 (68.0)
WBC ≥5 hpf or ≥10 mm^3^		
No	308 (95.1)	10 (20.0)
Yes	16 (4.9)	40 (80.0)
Non-*Escherichia coli* pathogen		
Not applicable	324 (100)	
No	—	46 (92.0)
Yes	—	4^b^ (8.0)

UTI, urinary tract infection.

^a^ UTI defined by growth of a uropathogen at ≥10,000 CFU/ml.

^b^ The non-*E. coli* pathogens were *Klebsiella* in three instances and *Enterococcus* in one instance.

**Figure 2:**
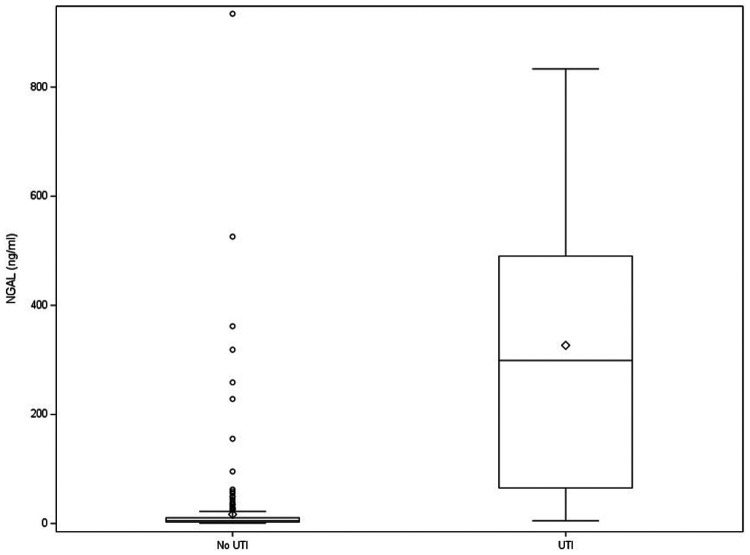
Box and whisker plot of urinary NGAL level in children with and without UTI. Dots represent outliers. Diamonds represent group means. NGAL, neutrophil gelatinase–associated lipocalin; UTI, urinary tract infection.

Biomarker levels, their respective AUC, and their respective sensitivity and specificity, which are determined using the cutoff that maximized the Youden index, are shown in Table 2. Urinary NGAL had the highest AUC (0.96; CI: 0.93–0.99), with a sensitivity of 90% (CI: 82–98) and specificity of 96% (CI: 93–98). Figure 2 shows NGAL levels in children with and without UTI. Three additional markers, IL-1β, CXCL1, and IL-8, also had AUCs greater than 0.85 and specificities greater than 76%. Supplement Table S3 shows the cutoff for each marker that maximized the Youden index with the corresponding sensitivity and specificity and the cutoff that minimized the distance to (0,1) on the ROC curve with the corresponding sensitivity and specificity.

**Table 2:** Univariate association between biomarkers and UTI in urine by decreasing accuracy (AUC).

Urinary biomarker	Number (no UTI/UTI)^a^	No UTI mean (SD) level	UTI mean (SD) level	*Q* value^b^	AUC (95% CI)	Sensitivity (95% CI)^c^	Specificity (95% CI)^c^
NGAL (ng/ml)	324/50	16.67 (67.98)	326.52 (258.56)	1.65 × 10^−51^	0.96 (0.93–0.99)	90% (82–98)	96% (93–98)
LE (≥trace)	324/50	NA	NA	6.56 × 10^−91^	0.93 (0.88–0.98)	88% (79–97)	96% (94–98)
WBC/mm^3^	270/44	3.25 (10.49)	73.36 (42.95)	3.49 × 10^−68^	0.91 (0.85–0.97)	77% (65–90)	98% (96–100)
IL-1β	323/48	201.55 (1,949.44)	2,430.62 (5,599.28)	5.77 × 10^−07^	0.89 (0.83–0.95)	85% (75–95)	82% (78–87)
CXCL1	323/49	74.70 (578.43)	1,510.65 (2,421.28)	3.28 × 10^−17^	0.89 (0.82–0.95)	82% (71–92)	88% (84–91)
IL-8	323/49	191.82 (2,135.17)	1,791.85 (2,844.06)	9.64 × 10^−06^	0.88 (0.81–0.94)	88% (79–97)	76% (72–81)
IL-6	323/49	409.61 (2,495.60)	6,733.33 (16,114.13)	9.02 × 10^−10^	0.83 (0.76–0.90)	65% (52–79)	89% (85–92)
IL-1α	323/49	6.25 (32.15)	60.43 (124.93)	1.12 × 10^−09^	0.83 (0.76–0.90)	78% (66–89)	85% (82–89)
CCL3	323/49	34.81 (175.71)	283.89 (564.08)	6.10 × 10^−09^	0.81 (0.73–0.89)	67% (54–80)	85% (81–89)
IL-17A	323/49	51.27 (187.29)	294.12 (718.46)	1.76 × 10^−06^	0.77 (0.71–0.84)	88% (79–97)	57% (51–62)
CCL4	323/49	546.12 (3,087.69)	3,118.81 (5,834.17)	8.64 × 10^−06^	0.77 (0.69–0.85)	69% (56–82)	77% (73–82)
MCP-1	323/49	3,778.30 (3,888.37)	12,713.19 (11,585.84)	6.34 × 10^−22^	0.74 (0.65–0.84)	59% (45–73)	90% (86–93)
IL-10	323/49	7.80 (31.45)	81.58 (251.27)	1.76 × 10^−06^	0.73 (0.65–0.81)	59% (45–73)	80% (76–85)
IL-21	323/49	22.27 (52.61)	131.29 (305.27)	3.27 × 10^−08^	0.71 (0.62–0.79)	57% (43–71)	82% (78–86)
GM-CSF	323/49	153.97 (281.16)	357.10 (726.76)	6.10 × 10^−04^	0.69 (0.62–0.76)	65% (52–79)	68% (63–73)
TNF-α	323/49	36.51 (61.18)	132.42 (296.08)	1.17 × 10^−06^	0.68 (0.59–0.76)	65% (52–79)	70% (65–75)
IL-5	322/49	74.61 (240.09)	225.13 (675.15)	4.23 × 10^−03^	0.67 (0.59–0.76)	59% (45–73)	74% (69–79)
IL-15	323/49	48.77 (89.53)	142.74 (452.23)	1.27 × 10^−03^	0.67 (0.60–0.74)	80% (68–91)	57% (52–62)
IL-27	323/49	477.96 (902.97)	1,309.41 (3,100.76)	2.44 × 10^−04^	0.67 (0.59–0.75)	71% (59–84)	66% (61–71)
IL-12p70	323/49	7.26 (52.79)	31.98 (130.55)	2.27 × 10^−02^	0.67 (0.58–0.75)	78% (66–89)	53% (48–59)
RANTES	323/49	46.30 (70.88)	162.20 (407.46)	8.64 × 10^−06^	0.66 (0.58–0.74)	49% (35–63)	75% (70–79)
IL-2	323/49	64.16 (86.09)	193.12 (559.57)	2.44 × 10^−04^	0.65 (0.57–0.73)	69% (56–82)	62% (57–68)
IL-4	323/49	49.80 (95.59)	110.05 (196.04)	9.09 × 10^−04^	0.65 (0.57–0.72)	84% (73–94)	45% (40–51)
IL-9	322/49	18.50 (35.51)	94.86 (361.52)	4.35 × 10^−04^	0.64 (0.57–0.72)	71% (59–84)	56% (50–61)
IP-10	323/49	4,069.67 (22,056.13)	10,692.07 (31,208.87)	7.21 × 10^−02^	0.64 (0.56–0.72)	55% (41–69)	69% (64–74)
IL-31	323/49	38.36 (106.99)	298.51 (1,201.70)	2.89 × 10^−04^	0.64 (0.56–0.72)	65% (52–79)	61% (56–67)
CXCL12	323/49	1,672.28 (2,313.05)	4,433.99 (7,200.54)	5.77 × 10^−07^	0.64 (0.55–0.73)	69% (56–82)	57% (52–63)
IL-1RA	322/49	160,439.60 (202,038.08)	388,807.18 (997,555.36)	4.53 × 10^−04^	0.61 (0.52–0.71)	43% (29–57)	83% (78–87)
IL-23	323/49	27.47 (84.31)	239.92 (1,024.03)	4.53 × 10^−04^	0.61 (0.52–0.69)	49% (35–63)	75% (70–80)
IL-13	323/49	23.48 (30.30)	36.12 (63.48)	2.86 × 10^−02^	0.60 (0.53–0.69)	69% (56–82)	54% (48–59)
IL-22	323/49	44.39 (129.82)	228.93 (1,107.42)	5.24 × 10^−03^	0.59 (0.51–0.67)	55% (41–69)	64% (59–69)
Eotaxin	323/49	83.58 (124.64)	158.63 (261.98)	1.62 × 10^−03^	0.59 (0.51–0.67)	63% (50–77)	54% (48–59)
IL-7	323/49	41.87 (60.72)	57.07 (89.91)	1.33 × 10^−01^	0.59 (0.51–0.67)	80% (68–91)	44% (39–50)
IFN-γ	323/49	532.17 (937.40)	538.05 (840.31)	9.67 × 10^−01^	0.55 (0.47–0.64)	76% (63–88)	41% (36–47)
IL-18	323/49	2,573.59 (4,153.73)	1,541.92 (1,849.57)	9.28 × 10^−02^	0.54 (0.46–0.62)	84% (73–94)	31% (26–36)
TNF-β	323/49	94.69 (347.80)	256.61 (1,142.01)	4.99 × 10^−02^	0.53 (0.45–0.61)	29% (16–41)	78% (74–83)
IFN-α	323/49	6.78 (24.09)	19.04 (80.26)	3.42 × 10^−02^	0.49 (0.40–0.57)	31% (18–44)	79% (75–83)

UTI, urinary tract infection; NGAL, neutrophil gelatinase–associated lipocalin; LE, leukocyte esterase.

Thresholds for the top four markers, NGAL, IL-1β, CXCL1, and IL-8, were 39.93, 65.79, 53.52, and 25.15, respectively. Levels are in pg/ml except for NGAL which was measured in ng/ml.

^a^ Not all markers were measured in all children, hence the slight differences in the counts.

^b^ *Q* values represent *p-*values corrected for multiple comparisons.

^c^ Sensitivity and specificity for the diagnosis of UTI in children. These values were determined using cutoffs that maximized the Youden index (sensitivity + specificity − 1).

In children with UTI, *E. coli* was the most frequently isolated organism (92%) on conventional urine culture*.* Urinary NGAL levels did not differ significantly (*p* = 0.35) between children with non-*E. coli* UTIs and those with *E. coli* UTIs (75.0% and 91.3%, respectively, had urinary NGAL ≥39.9 ng/ml). In contrast, patients with non-*E. coli* UTIs had significantly lower LE values compared to those of patients with *E. coli* UTIs (LE ≥ trace in 25.0% vs. 93.5%, respectively, *p* = 0.004).

No association existed between any of the covariates and the levels of the top four biomarkers examined, i.e., NGAL, IL-1β, CXCL1, and IL-8. Combining markers did not improve accuracy; the best combination, which used four variables, had a sensitivity of 0.90 and a specificity of 0.98 (AUC = 0.94; CI: 0.90–0.99). Pearson correlation coefficients between duplicates were greater than 0.99 for urinary NGAL, IL-1β, CXCL1, and IL-8.

## Discussion

We found that urinary NGAL had a sensitivity and specificity of 90% (CI: 82–98) and 96% (CI: 93–98) in differentiating UTI from no UTI in febrile young children with suspected UTI. In comparison, leukocyte esterase in this study had a sensitivity of 88% (CI: 79–97) and specificity of 96% (CI: 94–98). Urinary NGAL is involved in sequestering iron required for bacterial growth within the urinary tract and is released from neutrophils and intercalated cells in the renal collecting duct in response to infection or injury (12). A number of studies (4, 13–16) and a recent meta-analysis (10) have found that urinary NGAL levels differ in children with and without UTI, which is consistent with our results.

In 1,000 children presenting with UTI, 100 of whom are assumed to have a UTI (based on the prevalence of UTI in more recent studies that use an algorithm to select children for testing), two fewer UTIs would have been missed. Given the higher costs of the urinary NGAL test, this marginal difference supports the continued use of the LE as the screening test for UTI. However, certain other factors need to be considered. First, our results differ from the results of a recent meta-analysis comparing urinary NGAL and the leukocyte esterase test (10). In that meta-analysis, urinary NGAL was found to be more accurate than in our current study. Conversely, LE was less accurate in the meta-analysis than in our current study. This could be due to the different spectrum of patients enrolled (e.g., febrile) or different urine collection methods. Second, although the number of children with non-*E. coli* UTI was small in the current study, our data suggest that urinary NGAL may be a better screening test for UTI due to pathogens other than E. coli. Third, in children with ongoing bladder inflammation, such as children with spina bifida, the tests may perform differently (17). For these reasons, further research on the potential of urinary NGAL is warranted.

We found that IL-8 was significantly elevated in children with UTI (9, 18). IL-8, which is secreted by the urothelium, plays a major role in the recruitment of neutrophils to the urinary tract (19). IL-8 levels have been shown to be elevated in adults and children with UTI (20–22). However, we found that the accuracy of IL-8 was lower than that of LE, which limits its clinical utility.

We also found that CXCL1 (also known as GRO-α) was elevated significantly in children with UTI. Like IL-8, CXCL1 is also involved in neutrophil recruitment (8). CXCL1 is produced by the urothelium, particularly in response to uropathogenic *E. coli* exposure (23). CXCL1 has been shown to be elevated in mouse urine with uropathogenic *E. coli* exposure (24), and some authors have found increased levels of CXCL1 in humans with UTI (25). As with IL-8, we found that the accuracy of CXCL-1 was lower than LE, which limits its clinical utility.

We found that IL-1β was elevated in children with UTI. IL-1β is a pro-inflammatory biomarker that is secreted by macrophages in response to infection and serves to recruit neutrophils and other leukocytes. IL-1β is released very early in response to infection (26, 27). IL-1β has been shown to be elevated in children with cystitis (28) and acute pyelonephritis (27, 29). However, IL-1β has not been studied as a screening biomarker for febrile UTIs in children. Our results indicate that IL-1β as a biomarker to detect UTI in febrile children deserves more study.

In a previous study (30), we reported that screening using the leukocyte esterase test could miss a significant proportion of infections caused by organisms other than *E. coli*. The current study, while small, seems to reinforce those findings; of the four children with infections caused by organisms other than *E. coli*, only 1 had a positive LE test, whereas three had high (≥39.9 ng/ml) urinary NGAL levels. Further research is needed to establish whether urinary NGAL could serve as an accurate marker for UTI secondary to organisms other than *E. coli*.

This study has several limitations. In order to allow us to compare the accuracy of various biomarkers to the LE test, we did not require a positive LE test in our definition of UTI. Thus, the misclassification of children with asymptomatic bacteriuria without pyuria as UTI is a potential limitation. Nevertheless, asymptomatic bacteriuria masquerading as UTI is highly unlikely because all children included were symptomatic (all were tested and treated for UTI) and because, based on the prevalence of asymptomatic bacteria without pyuria in this population (i.e., 0.21%) (31), no more than one child included in this sample likely has asymptomatic bacteriuria without pyuria. A second source of misclassification of children may have come from contaminated samples read as having UTI. We attempted to minimize this by only using catheterized samples.

Our findings further support the use of urinary NGAL as a screening test for UTI. More research regarding the utility of measuring urinary NGAL, especially in children with non-*E. coli* UTI and in children with spina bifida in whom the performance of the leukocyte esterase test is suboptimal, is needed.

## Data Availability

The original contributions presented in the study are included in the article/Supplementary Material, further inquiries can be directed to the corresponding author.

## Supplementary material

The Supplementary Material for this article can be found online at: https://www.frontiersin.org/articles/10.3389/fped.2023.1163546/full#supplementary-material .
