# Supplementary material for: Biomarkers for febrile urinary tract infection in children
Source: Front Pediatr. 2023 May 9;11:1163546. doi: 10.3389/fped.2023.1163546 (PMC10203466; doi:10.3389/fped.2023.1163546)
Supplement: Supplementary file 1 [file Table1.docx]

**Supplement**

**Table 3**. Sensitivity and specificity of biomarkers in screening for urinary tract infection (UTI) using two different methods of choosing a cutoff values

|  |  | **J cutoff (maximizes Youden’s index)^b^** | | | | |  | **D cutoff (minimizes distance to perfect accuracy)^c^** | | | | |
| --- | --- | --- | --- | --- | --- | --- | --- | --- | --- | --- | --- | --- |
| **Urinary**  **Biomarker** | **AUC^a^** | **Cutoff** | **Sensitivity (95% CI)** | | **Specificity (95% CI)** | |  | **Cutoff** | **Sensitivity (95% CI)** | | **Specificity (95% CI)** | |
|  |  |  |  |  |  |  |  |  |  |  |  |  |
| **NGAL (ng/mL)** | 0.96 | 39.93 | 90% | (82-98) | 96% | (93-98) |  | 39.93 | 90% | (82-98) | 96% | (93-98) |
| **LE (≥trace)** | 0.93 | 2.00 | 88% | (79-97) | 96% | (94-98) |  | 2.00 | 88% | (79-97) | 96% | (94-98) |
| **WBC/mm^3^** | 0.91 | 14.00 | 77% | (65-90) | 98% | (96-100) |  | 14.00 | 77% | (65-90) | 98% | (96-100) |
| **IL-1-β** | 0.89 | 65.79 | 85% | (75-95) | 82% | (78-87) |  | 65.79 | 85% | (75-95) | 82% | (78-87) |
| **CXCL1** | 0.89 | 53.52 | 82% | (71-92) | 88% | (84-91) |  | 53.52 | 82% | (71-92) | 88% | (84-91) |
| **IL-8** | 0.88 | 25.15 | 88% | (79-97) | 76% | (72-81) |  | 34.70 | 84% | (73-94) | 80% | (76-85) |
| **IL-6** | 0.83 | 570.37 | 65% | (52-79) | 89% | (85-92) |  | 252.34 | 76% | (63-88) | 76% | (72-81) |
| **IL-1-α** | 0.83 | 1.53 | 78% | (66-89) | 85% | (82-89) |  | 1.33 | 80% | (68-91) | 83% | 79-87) |
| **CCL3** | 0.81 | 38.61 | 67% | (54-80) | 85% | (81-89) |  | 24.24 | 76% | (63-88) | 76% | (72-81) |
| **IL-17A** | 0.77 | 11.41 | 88% | (79-97) | 57% | (51-62) |  | 35.29 | 67% | (54-80) | 74% | (69-79) |
| **CCL4** | 0.77 | 365.72 | 69% | (56-82) | 77% | (73-82) |  | 365.72 | 69% | (56-82) | 77% | (73-82) |
| **MCP-1** | 0.74 | 7338.55 | 59% | (45-73) | 90% | (86-93) |  | 4673.91 | 69% | (56-82) | 71% | (66-76) |
| **IL-10** | 0.73 | 5.24 | 59% | (45-73) | 80% | (76-85) |  | 4.00 | 67% | (54-80) | 72% | (70-77) |
| **IL-21** | 0.71 | 34.40 | 57% | (43-71) | 82% | (78-86) |  | 34.40 | 57% | (43-71) | 82% | (78-86) |
| **GM-CSF** | 0.69 | 99.01 | 65% | (52-79) | 68% | (63-73) |  | 99.01 | 65% | (52-79) | 68% | (63-73) |
| **TNF-α** | 0.68 | 31.05 | 65% | (52-79) | 70% | (65-75) |  | 31.05 | 65% | (52-79) | 70% | (65-75) |
| **IL-5** | 0.67 | 59.22 | 59% | (45-73) | 74% | (69-79) |  | 50.00 | 63% | (50-77) | 69% | (64-74) |
| **IL-15** | 0.67 | 14.79 | 80% | (68-91) | 57% | (52-62) |  | 16.90 | 71% | (59-84) | 62% | (57-68) |
| **IL-27** | 0.67 | 212.04 | 71% | (59-84) | 66% | (61-71) |  | 212.04 | 71% | (59-84) | 66% | (61-71) |
| **IL-12p70** | 0.67 | 3.72 | 78% | (66-89) | 53% | (48-59) |  | 5.23 | 61% | (48-75) | 68% | (63-73) |
| **RANTES** | 0.66 | 47.35 | 49% | (35-63) | 75% | (70-79) |  | 35.24 | 57% | (43-71) | 66% | (60-71) |
| **IL-2** | 0.65 | 44.72 | 69% | (56-82) | 62% | (57-68) |  | 44.72 | 69% | (56-82) | 62% | (57-68) |
| **IL-4** | 0.65 | 5.61 | 84% | (73-94) | 45% | (40-51) |  | 19.29 | 69% | (56-82) | 54% | (49-60) |
| **IL-9** | 0.64 | 7.91 | 71% | (59-84) | 56% | (50-61) |  | 7.91 | 71% | (59-84) | 56% | (50-61) |
| **IP-10** | 0.64 | 998.94 | 55% | (41-69) | 69% | (64-74) |  | 998.94 | 55% | (41-69) | 69% | (64-74) |
| **IL-31** | 0.64 | 1.13 | 65% | (52-79) | 61% | (56-67) |  | 1.13 | 65% | (52-79) | 61% | (56-67) |
| **CXCL12** | 0.64 | 977.63 | 69% | (56-82) | 57% | (52-63) |  | 1042.34 | 67% | (54-80) | 59% | (53-64) |
| **IL-1RA** | 0.61 | 218441.83 | 43% | (29-57) | 83% | (78-87) |  | 111367.04 | 76% | (63-88) | 49% | (43-54) |
| **IL-23** | 0.61 | 22.31 | 49% | (35-63) | 75% | (70-80) |  | 22.31 | 49% | (35-63) | 75% | (70-80) |
| **IL-13** | 0.60 | 13.32 | 69% | (56-82) | 54% | (48-59) |  | 13.32 | 69% | (56-82) | 54% | (48-59) |
| **IL-22** | 0.59 | 3.48 | 55% | (41-69) | 64% | (59-69) |  | 0.01 | 57% | (43-71) | 62% | (56-67) |
| **Eotaxin** | 0.59 | 39.04 | 63% | (50-77) | 54% | (48-59) |  | 39.04 | 63% | (50-77) | 54% | (48-59) |
| **IL-7** | 0.59 | 13.69 | 80% | (68-91) | 44% | (39-50) |  | 16.25 | 69% | (56-82) | 50% | (45-56) |
| **IFN-γ** | 0.55 | 112.87 | 76% | (63-88) | 41% | (36-47) |  | 146.56 | 63% | (50-77) | 49% | (43-54) |
| **IL-18** | 0.54 | 2391.41 | 84% | (73-94) | 31% | (26-36) |  | 748.45 | 51% | (37-65) | 58% | (53-64) |
| **TNF-β** | 0.53 | 21.70 | 29% | (16-41) | 78% | (74-83) |  | 0.47 | 43% | (29-57) | 63% | (57-68) |
| **IFN-α** | 0.49 | 1.10 | 31% | (18-44) | 79% | (75-83) |  | 1.10 | 31% | (18-44) | 79% | (75-83) |

^a^Area under the ROC curve as a measure of accuracy.

^b^J, the Youden index, combines sensitivity and specificity into a single measure (sensitivity + specificity -1). The J cutoff is the level of the marker that maximizes J. Sensitivity and specificity for the diagnosis of UTI in children correspond to the J cutoff.

^c^D is the distance from the ROC curve to the 'perfect' point where 1-specificity=0 and sensitivity=1. The D cutoff is the level of the marker that minimizes D. Sensitivity and specificity for the diagnosis of UTI in children correspond to the D cutoff.
